# 3-Eth­oxy­carbonyl-2-hy­droxy-6-meth­oxy-4-methyl­benzoic acid

**DOI:** 10.1107/S1600536812015012

**Published:** 2012-04-18

**Authors:** Yun-Xia Deng, Tao Guo, Hui Xie, Sheng-Li Pan

**Affiliations:** a School of Pharmacy, Fudan University, Shanghai 201203, People’s Republic of China; b School of Life and Engineering, Lanzhou University of Technology, Lanzhou 730050, People’s Republic of China

## Abstract

The title compound, C_12_H_14_O_6_, a substituted isophthalic acid monoester which was isolated from the lichen *Thamnolia vermicularis* var. *subuliformis*, displays intra­molecular carbox­yl–meth­oxy O—H⋯O and hy­droxy–carboxyl O—H⋯O hydrogen-bonding inter­actions. The terminal methyl group of the ethyl ester is disordered over two sets of sites with occupancies of 0.599 (19) and 0.401 (19).

## Related literature

For general background to the phenol compounds isolated from the lichen *Thamnolia vermicularis* var.* subuliformis*, see: Jiang *et al.* (2002 ▶); Milenkovic-Andjelkovic (2010 ▶). For applications of analogs of the title compound, see: Huneck (1999 ▶).
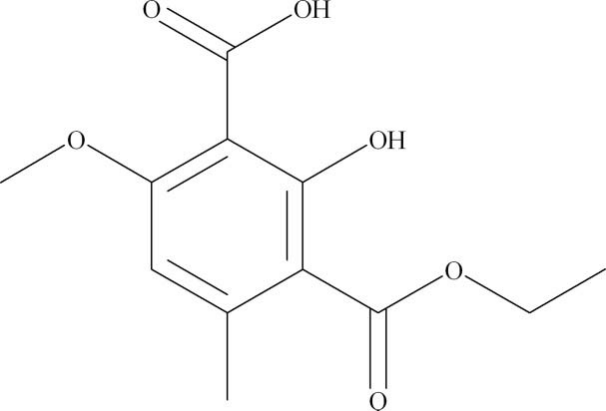


## Experimental

### 

#### Crystal data

C_12_H_14_O_6_
*M* *_r_* = 254.23
Triclinic, 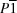

*a* = 6.8460 (14) Å
*b* = 8.0065 (16) Å
*c* = 11.469 (2) Å
α = 97.059 (4)°
β = 95.987 (4)°
γ = 98.072 (4)°
*V* = 612.9 (2) Å^3^
*Z* = 2
Mo *K*α radiation
μ = 0.11 mm^−1^
*T* = 293 K
0.39 × 0.30 × 0.11 mm

#### Data collection

Bruker SMART CCD area-detector diffractometer
Absorption correction: multi-scan (*SADABS*; Bruker, 2003 ▶) *T*_min_ = 0.245, *T*_max_ = 1.000
3352 measured reflections
2359 independent reflections
1379 reflections with *I* > 2σ(*I*)
*R*_int_ = 0.083

#### Refinement

*R*[*F* ^2^ > 2σ(*F* ^2^)] = 0.057
*wR*(*F* ^2^) = 0.174
*S* = 0.91
2359 reflections
186 parameters
22 restraints
H atoms treated by a mixture of independent and constrained refinement
Δρ_max_ = 0.32 e Å^−3^
Δρ_min_ = −0.21 e Å^−3^

### 

Data collection: *SMART* (Bruker, 2003 ▶); cell refinement: *SAINT* (Bruker, 2003 ▶); data reduction: *SAINT*; program(s) used to solve structure: *SHELXS97* (Sheldrick, 2008 ▶); program(s) used to refine structure: *SHELXL97* (Sheldrick, 2008 ▶); molecular graphics: *SHELXTL* (Sheldrick, 2008 ▶); software used to prepare material for publication: *SHELXTL*.

## Supplementary Material

Crystal structure: contains datablock(s) I, global. DOI: 10.1107/S1600536812015012/zs2195sup1.cif

Structure factors: contains datablock(s) I. DOI: 10.1107/S1600536812015012/zs2195Isup2.hkl

Supplementary material file. DOI: 10.1107/S1600536812015012/zs2195Isup3.cml

Additional supplementary materials:  crystallographic information; 3D view; checkCIF report

## Figures and Tables

**Table 1:** Hydrogen-bond geometry (Å, °)

*D*—H⋯*A*	*D*—H	H⋯*A*	*D*⋯*A*	*D*—H⋯*A*
O4—H4⋯O2	0.88 (2)	1.77 (3)	2.535 (3)	144 (4)
O1—H1⋯O3	0.84 (2)	1.78 (3)	2.524 (3)	146 (4)

**Fig. 1.**
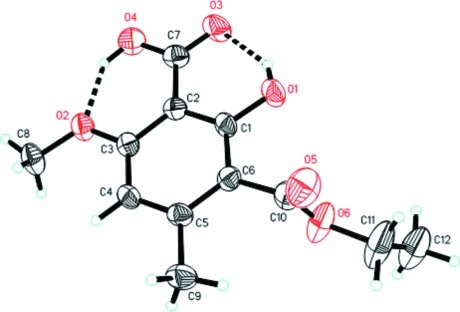
The molecular structure of the title compound, showing 30% probability displacement ellipsoids. The disorder in the ethyl ester group is not shown. Intramolecular hydrogen bonds are shown as dashed lines.

**Fig. 2.**
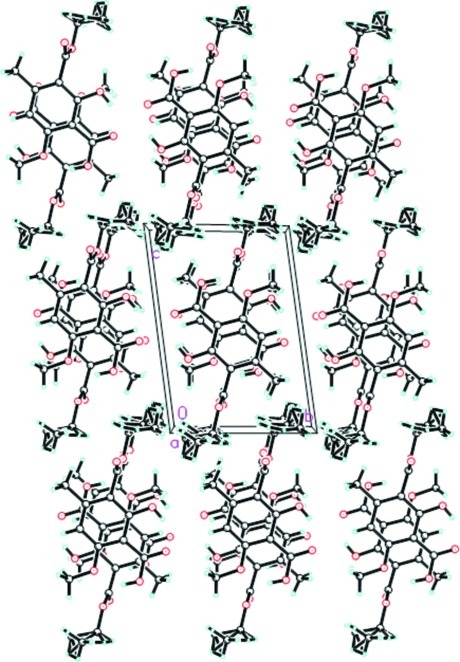
The crystal packing of the title compound viewed down the a axis.

## supplementary crystallographic information

### Comment

The title compound, C_12_H_14_O_6_, a substituted isophthalic acid monoester, is one of the phenol compounds isolated from the lichen *Thamnolia vermicularis* var.*subuliformis* (Jiang *et al.*, 2002; Milenkovic-Andjelkovic, 2010). The X-ray structural analysis of this compound reported here confirms the assignment of its structure determined from experimental spectroscopic data. In the molecule (Fig. 1), intramolecular carboxylic acid O—H···O_methoxy_ and hydroxy O—H···O_carboxyl_ hydrogen -bonding interactions (Table 1) result in the formation of two six-membered rings. In the crystal (Fig. 2), no significant hydrogen-bonding interactions are found. The terminal methyl group of the ethyl ester is disordered over two sites with occupancies 0.599:0.401.

### Experimental

Extraction of the title compound. The air-dried and powdered plant materials (5 kg) were extracted by 95% EtOH (3 times, 20*L*) at room temperature and concentrated under vacuum. The residue was partitioned with petroleum ether (PE) and EtOAc,successively. The EtOAc extract (47 g) was chromatographed on a silica gel column eluted successively with PE-EtOAc/ EtOAc–MeOH to afford six major fractions. Fraction 3 eluted with PE-EtOAc (1:3) was further purified by silica gel chromatography [CHCl_3_-MeOH (15:1)] and then Sephadex LH-20 using MeOH (100%) to yield the title compound (200 mg). The solvent was removed *in vacuo* to give colorless crystals (m.p. 435–437 K). ^1^*H*-NMR (CDCl_3_, 400 MHz): 12.6 (1H, s, OH), 11.2 (1H, s, COOH), 6.34 (1H, s), 4.4 (2H, q, CH_2_), 4.08 (3H, s, CH_3_), 2.37 (3H, s, CH_3_), 1.39 (3H, t, CH_3_). Crystals suitable for X-ray diffraction were obtained by slow evaporation of a methanol solution.

### Refinement

Hydroxy and carboxylic acid H-atoms were located in a difference-Fourier analysis and both positional and isotropic displacement parameters were refined. Other H-atoms were positioned geometrically with C—H = 0.93 Å (for aromatic H) or 0.96 or 0.97 Å (for methyl or methylene H-atoms respectively) and constrained to ride on their parent atoms, with *U*_iso_(H) = 1.2 or 1.5 *U*_eq_(C). Disorder in the terminal methyl group (C12) of the ethyl ester resulted in the refinement at two sites with occupancies of 0.599 (19) (C12) and 0.401 (19) (C12').

### Crystal data

**Table:**

C_12_H_14_O_6_	*Z* = 2
*M**_r_* = 254.23	*F*(000) = 268
Triclinic, *P*1	*D*_x_ = 1.378 Mg m^−^^3^
Hall symbol: -P 1	Melting point = 435–437 K
*a* = 6.8460 (14) Å	Mo *K*α radiation, λ = 0.71073 Å
*b* = 8.0065 (16) Å	Cell parameters from 969 reflections
*c* = 11.469 (2) Å	θ = 5.2–52.9°
α = 97.059 (4)°	µ = 0.11 mm^−^^1^
β = 95.987 (4)°	*T* = 293 K
γ = 98.072 (4)°	Prismatic, colorless
*V* = 612.9 (2) Å^3^	0.39 × 0.30 × 0.11 mm

### Data collection

**Table:**

Bruker SMART CCD area-detector diffractometer	2359 independent reflections
Radiation source: fine-focus sealed tube	1379 reflections with *I* > 2σ(*I*)
Graphite monochromator	*R*_int_ = 0.083
φ and ω scans	θ_max_ = 26.0°, θ_min_ = 1.8°
Absorption correction: multi-scan (*SADABS*; Bruker, 2003)	*h* = −8→8
*T*_min_ = 0.245, *T*_max_ = 1.000	*k* = −9→7
3352 measured reflections	*l* = −14→13

### Refinement

**Table:**

Refinement on *F*^2^	Secondary atom site location: difference Fourier map
Least-squares matrix: full	Hydrogen site location: inferred from neighbouring sites
*R*[*F*^2^ > 2σ(*F*^2^)] = 0.057	H atoms treated by a mixture of independent and constrained refinement
*wR*(*F*^2^) = 0.174	*w* = 1/[σ^2^(*F*_o_^2^) + (0.0955*P*)^2^] where *P* = (*F*_o_^2^ + 2*F*_c_^2^)/3
*S* = 0.91	(Δ/σ)_max_ < 0.001
2359 reflections	Δρ_max_ = 0.32 e Å^−^^3^
186 parameters	Δρ_min_ = −0.21 e Å^−^^3^
22 restraints	Extinction correction: *SHELXL97* (Sheldrick, 2008), Fc^*^=kFc[1+0.001xFc^2^λ^3^/sin(2θ)]^-1/4^
Primary atom site location: structure-invariant direct methods	Extinction coefficient: 0.036 (12)

### Special details

**Table:**

Geometry. All e.s.d.'s (except the e.s.d. in the dihedral angle between two l.s. planes) are estimated using the full covariance matrix. The cell e.s.d.'s are taken into account individually in the estimation of e.s.d.'s in distances, angles and torsion angles; correlations between e.s.d.'s in cell parameters are only used when they are defined by crystal symmetry. An approximate (isotropic) treatment of cell e.s.d.'s is used for estimating e.s.d.'s involving l.s. planes.
Refinement. Refinement of *F*^2^ against ALL reflections. The weighted *R*-factor *wR* and goodness of fit *S* are based on *F*^2^, conventional *R*-factors *R* are based on *F*, with *F* set to zero for negative *F*^2^. The threshold expression of *F*^2^ > σ(*F*^2^) is used only for calculating *R*-factors(gt) *etc*. and is not relevant to the choice of reflections for refinement. *R*-factors based on *F*^2^ are statistically about twice as large as those based on *F*, and *R*- factors based on ALL data will be even larger.

### Fractional atomic coordinates and isotropic or equivalent isotropic displacement parameters (Å^2^)

**Table:**

	*x*	*y*	*z*	*U*_iso_*/*U*_eq_	Occ. (<1)
O1	0.1285 (4)	0.1888 (3)	0.3537 (2)	0.0692 (7)	
O2	0.3292 (3)	0.6477 (3)	0.66405 (18)	0.0640 (7)	
O3	0.1945 (4)	0.1389 (3)	0.5664 (2)	0.0771 (8)	
O4	0.2762 (4)	0.3453 (3)	0.7124 (2)	0.0773 (8)	
O5	−0.0464 (4)	0.3799 (4)	0.1320 (2)	0.0992 (10)	
O6	0.2714 (5)	0.3547 (4)	0.1310 (2)	0.0992 (10)	
C1	0.1806 (4)	0.3544 (3)	0.3938 (3)	0.0501 (8)	
C2	0.2325 (4)	0.4167 (3)	0.5141 (2)	0.0472 (7)	
C3	0.2804 (4)	0.5934 (4)	0.5465 (2)	0.0485 (7)	
C4	0.2788 (4)	0.7035 (3)	0.4632 (3)	0.0518 (8)	
H4A	0.3110	0.8204	0.4867	0.062*	
C5	0.2289 (4)	0.6395 (4)	0.3439 (3)	0.0526 (8)	
C6	0.1793 (4)	0.4666 (4)	0.3092 (2)	0.0519 (8)	
C7	0.2327 (4)	0.2914 (4)	0.5983 (3)	0.0575 (8)	
C8	0.3758 (6)	0.8247 (4)	0.7062 (3)	0.0777 (11)	
H8A	0.4967	0.8708	0.6783	0.117*	
H8B	0.3928	0.8415	0.7912	0.117*	
H8C	0.2696	0.8814	0.6777	0.117*	
C9	0.2292 (6)	0.7624 (4)	0.2541 (3)	0.0742 (10)	
H9A	0.3583	0.8302	0.2612	0.111*	
H9B	0.1317	0.8355	0.2682	0.111*	
H9C	0.1977	0.6999	0.1759	0.111*	
C10	0.1172 (6)	0.3942 (4)	0.1827 (3)	0.0668 (9)	
C11	0.2310 (10)	0.2902 (7)	0.0054 (4)	0.136 (2)	
H11A	0.1002	0.2215	−0.0116	0.164*	0.599 (19)
H11B	0.2327	0.3847	−0.0402	0.164*	0.599 (19)
H11C	0.3379	0.3382	−0.0339	0.164*	0.401 (19)
H11D	0.1099	0.3252	−0.0259	0.164*	0.401 (19)
C12	0.376 (2)	0.1907 (18)	−0.0273 (7)	0.128 (5)	0.599 (19)
H12A	0.3768	0.0993	0.0196	0.192*	0.599 (19)
H12B	0.5047	0.2605	−0.0141	0.192*	0.599 (19)
H12C	0.3461	0.1445	−0.1096	0.192*	0.599 (19)
C12'	0.213 (4)	0.107 (2)	−0.0170 (14)	0.137 (7)	0.401 (19)
H12D	0.2147	0.0720	−0.0999	0.205*	0.401 (19)
H12E	0.0899	0.0571	0.0065	0.205*	0.401 (19)
H12F	0.3219	0.0709	0.0276	0.205*	0.401 (19)
H1	0.144 (7)	0.131 (5)	0.410 (3)	0.113 (16)*	
H4	0.301 (6)	0.457 (3)	0.728 (4)	0.110 (16)*	

### Atomic displacement parameters (Å^2^)

**Table:**

	*U*^11^	*U*^22^	*U*^33^	*U*^12^	*U*^13^	*U*^23^
O1	0.0993 (18)	0.0384 (13)	0.0710 (16)	0.0188 (11)	0.0075 (13)	0.0049 (10)
O2	0.0797 (15)	0.0543 (14)	0.0555 (14)	0.0149 (10)	−0.0031 (10)	0.0023 (9)
O3	0.1013 (19)	0.0482 (15)	0.0865 (18)	0.0195 (12)	0.0037 (13)	0.0254 (12)
O4	0.106 (2)	0.0709 (18)	0.0605 (16)	0.0252 (15)	0.0042 (13)	0.0245 (13)
O5	0.091 (2)	0.126 (3)	0.0735 (18)	0.0161 (17)	−0.0105 (15)	0.0056 (15)
O6	0.124 (2)	0.132 (3)	0.0519 (15)	0.069 (2)	0.0086 (14)	−0.0027 (13)
C1	0.0554 (17)	0.0362 (16)	0.0614 (19)	0.0177 (13)	0.0087 (13)	0.0047 (12)
C2	0.0432 (16)	0.0471 (17)	0.0572 (18)	0.0180 (12)	0.0096 (13)	0.0148 (13)
C3	0.0434 (16)	0.0484 (17)	0.0553 (18)	0.0148 (12)	0.0039 (12)	0.0065 (13)
C4	0.0540 (18)	0.0389 (16)	0.0638 (19)	0.0112 (12)	0.0069 (14)	0.0073 (13)
C5	0.0536 (18)	0.0469 (17)	0.063 (2)	0.0151 (13)	0.0113 (14)	0.0168 (14)
C6	0.0583 (18)	0.0502 (18)	0.0519 (18)	0.0197 (14)	0.0114 (14)	0.0091 (13)
C7	0.0561 (18)	0.055 (2)	0.069 (2)	0.0188 (14)	0.0095 (15)	0.0221 (15)
C8	0.096 (3)	0.056 (2)	0.072 (2)	0.0133 (18)	−0.0136 (19)	−0.0104 (16)
C9	0.093 (3)	0.061 (2)	0.072 (2)	0.0127 (18)	0.0080 (18)	0.0270 (17)
C10	0.086 (3)	0.058 (2)	0.060 (2)	0.0230 (18)	0.0068 (19)	0.0108 (15)
C11	0.199 (5)	0.168 (5)	0.055 (3)	0.097 (5)	0.006 (3)	−0.006 (3)
C12	0.173 (10)	0.159 (9)	0.068 (5)	0.083 (8)	0.021 (5)	0.005 (5)
C12'	0.163 (13)	0.131 (11)	0.107 (9)	0.037 (9)	−0.003 (8)	−0.020 (7)

### Geometric parameters (Å, º)

**Table:**

O1—C1	1.334 (3)	C6—C10	1.488 (4)
O1—H1	0.84 (2)	C8—H8A	0.9600
O2—C3	1.357 (3)	C8—H8B	0.9600
O2—C8	1.420 (4)	C8—H8C	0.9600
O3—C7	1.214 (4)	C9—H9A	0.9600
O4—C7	1.316 (4)	C9—H9B	0.9600
O4—H4	0.88 (2)	C9—H9C	0.9600
O5—C10	1.190 (4)	C11—C12	1.411 (10)
O6—C10	1.321 (4)	C11—C12'	1.441 (15)
O6—C11	1.453 (4)	C11—H11A	0.9700
C1—C2	1.399 (4)	C11—H11B	0.9700
C1—C6	1.401 (4)	C11—H11C	0.9580
C2—C3	1.401 (4)	C11—H11D	0.9607
C2—C7	1.475 (4)	C12—H12A	0.9600
C3—C4	1.377 (4)	C12—H12B	0.9600
C4—C5	1.390 (4)	C12—H12C	0.9600
C4—H4A	0.9300	C12'—H12D	0.9600
C5—C6	1.376 (4)	C12'—H12E	0.9600
C5—C9	1.509 (4)	C12'—H12F	0.9600
			
C1—O1—H1	110 (3)	C5—C9—H9A	109.5
C3—O2—C8	120.1 (2)	C5—C9—H9B	109.5
C7—O4—H4	113 (3)	H9A—C9—H9B	109.5
C10—O6—C11	115.9 (3)	C5—C9—H9C	109.5
O1—C1—C2	122.7 (2)	H9A—C9—H9C	109.5
O1—C1—C6	116.8 (3)	H9B—C9—H9C	109.5
C2—C1—C6	120.5 (3)	O5—C10—O6	123.6 (3)
C1—C2—C3	117.9 (2)	O5—C10—C6	125.6 (3)
C1—C2—C7	117.7 (3)	O6—C10—C6	110.8 (3)
C3—C2—C7	124.5 (3)	C12—C11—O6	109.7 (5)
O2—C3—C4	122.7 (3)	C12'—C11—O6	112.2 (7)
O2—C3—C2	115.7 (2)	C12—C11—H11A	109.7
C4—C3—C2	121.6 (3)	O6—C11—H11A	109.7
C3—C4—C5	119.8 (3)	C12—C11—H11B	109.7
C3—C4—H4A	120.1	O6—C11—H11B	109.7
C5—C4—H4A	120.1	H11A—C11—H11B	108.2
C6—C5—C4	120.1 (2)	C12'—C11—H11C	108.5
C6—C5—C9	121.0 (3)	O6—C11—H11C	108.9
C4—C5—C9	118.9 (3)	C12'—C11—H11D	109.6
C5—C6—C1	120.2 (3)	O6—C11—H11D	108.5
C5—C6—C10	121.5 (3)	H11C—C11—H11D	109.1
C1—C6—C10	118.3 (3)	C11—C12—H12A	109.5
O3—C7—O4	118.1 (3)	C11—C12—H12B	109.5
O3—C7—C2	122.5 (3)	C11—C12—H12C	109.5
O4—C7—C2	119.4 (3)	C11—C12'—H12D	109.5
O2—C8—H8A	109.5	C11—C12'—H12E	109.5
O2—C8—H8B	109.5	H12D—C12'—H12E	109.5
H8A—C8—H8B	109.5	C11—C12'—H12F	109.5
O2—C8—H8C	109.5	H12D—C12'—H12F	109.5
H8A—C8—H8C	109.5	H12E—C12'—H12F	109.5
H8B—C8—H8C	109.5		
			
O1—C1—C2—C3	−178.6 (2)	C9—C5—C6—C10	−2.2 (5)
C6—C1—C2—C3	0.6 (4)	O1—C1—C6—C5	179.2 (3)
O1—C1—C2—C7	1.4 (4)	C2—C1—C6—C5	−0.1 (4)
C6—C1—C2—C7	−179.4 (2)	O1—C1—C6—C10	1.1 (4)
C8—O2—C3—C4	1.8 (4)	C2—C1—C6—C10	−178.2 (3)
C8—O2—C3—C2	−178.6 (3)	C1—C2—C7—O3	1.7 (4)
C1—C2—C3—O2	179.8 (2)	C3—C2—C7—O3	−178.3 (3)
C7—C2—C3—O2	−0.2 (4)	C1—C2—C7—O4	−178.4 (3)
C1—C2—C3—C4	−0.5 (4)	C3—C2—C7—O4	1.5 (4)
C7—C2—C3—C4	179.5 (2)	C11—O6—C10—O5	−0.3 (6)
O2—C3—C4—C5	179.5 (2)	C11—O6—C10—C6	−177.2 (3)
C2—C3—C4—C5	−0.1 (4)	C5—C6—C10—O5	−83.8 (4)
C3—C4—C5—C6	0.6 (4)	C1—C6—C10—O5	94.2 (4)
C3—C4—C5—C9	−179.7 (3)	C5—C6—C10—O6	93.0 (4)
C4—C5—C6—C1	−0.5 (4)	C1—C6—C10—O6	−88.9 (4)
C9—C5—C6—C1	179.8 (3)	C10—O6—C11—C12	−156.0 (9)
C4—C5—C6—C10	177.5 (3)	C10—O6—C11—C12'	−100.3 (13)

### Hydrogen-bond geometry (Å, º)

**Table:**

*D*—H···*A*	*D*—H	H···*A*	*D*···*A*	*D*—H···*A*
O4—H4···O2	0.88 (2)	1.77 (3)	2.535 (3)	144 (4)
O1—H1···O3	0.84 (2)	1.78 (3)	2.524 (3)	146 (4)
